# Long undecoded transcript isoform (LUTI) detection in meiotic budding yeast by direct RNA and transcript leader sequencing

**DOI:** 10.1016/j.xpro.2022.101145

**Published:** 2022-02-04

**Authors:** Amy Tresenrider, Minghao Chia, Folkert J. van Werven, Elçin Ünal

**Affiliations:** 1 Department of Molecular and Cell Biology, Barker Hall, University of California, Berkeley, Berkeley, CA 94720, USA; 2 Genome Institute of Singapore, 60 Biopolis Street, Genome, #02-01, Singapore 138672, Singapore; 3 Department of Genome Sciences, Foege Hall, University of Washington, Seattle, WA 98105, USA; 4 The Francis Crick Institute, 1 Midland Road, NW1 1AT London, UK

**Keywords:** Bioinformatics, Sequence analysis, Genetics, Genomics, Sequencing, Model Organisms, Molecular Biology

## Abstract

LUTIs (Long Undecoded Transcript Isoforms) are 5′-extended and poorly translated mRNAs that can downregulate transcription from promoters more proximal to a gene’s coding sequence (CDS). In this protocol, polyA RNA is extracted from budding yeast cells undergoing highly synchronized meiosis. Using a combination of long-read direct RNA sequencing and transcript leader sequencing (TL-seq), meiosis-specific LUTIs are systematically identified. Following identification, TL-seq is used to quantify the abundance of both LUTI and the more canonical gene-proximal (PROX) transcripts.

For complete details on the use and execution of this protocol, please refer to Tresenrider et al. (2021).

## Highlights

• A protocol for the detection of Long Undecoded Transcript Isoforms (LUTIs) in yeast
• Applies direct RNA sequencing and TL-seq to annotate LUTIs
• Quantification of LUTIs and canonical mRNAs expressed from the same gene locus

## Graphical abstract

**Figure:**
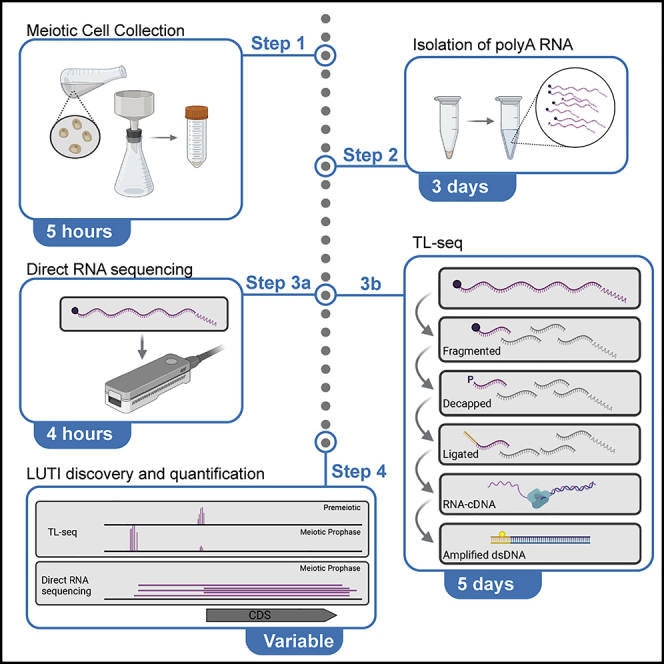

## Before you begin

Deep sequencing and tiling array-based technologies have previously identified hundreds of 5′-extended transcripts during the budding yeast meiotic program (Lardenois et al., 2011, 2015; Brar et al., 2012; Kim Guisbert et al., 2012). Subsequently, an in-depth investigation at the *NDC80* locus revealed that a 5′-extended transcript produced little to no Ndc80 protein (Chen et al., 2017). This was due to upstream open reading frames (uORFs) which engaged with the ribosome and prevented it from scanning to the *NDC80* CDS start site. It was with this knowledge that poorly translated and 5′-extended transcripts earned the title of LUTIs, for Long Undecoded Transcript Isoforms. At the *NDC80* locus, transcription of the LUTI also repressed transcription initiation from the canonical promoter through alterations to the chromatin (Chia et al., 2017). Almost 400 instances whereby LUTI expression correlates poorly and sometimes negatively with the level of translation (ribosome profiling) or protein level (mass spectrometry) during yeast meiosis have since been identified (Cheng et al., 2018). However, there was no previous method that systematically identified all LUTIs, regardless of their correlation with translation or protein abundance.

This method outlines how to identify all condition-specific LUTIs and for the first time how to quantify both the LUTI and canonical transcript isoforms, referred to as PROX isoforms due to their gene-proximal transcription start sites (TSS) when compared to LUTIs. The protocol is designed specifically for *S. cerevisiae* strains of the SK1 background that harbor two key meiotic entry regulators, *IME1* and *IME4*, under a copper inducible promoter (originally described in (Berchowitz et al., 2013)). Cells are collected at a premeiotic stage and during meiotic prophase.

### Preparation of cells for meiosis

**Timing: 3 days**

In this step, cells are prepared for meiotic induction as described in Chia and van Werven (2016).

1. Construct or obtain yeast strains in which the promoters for *IME1* and *IME4* are replaced with the *CUP1* promoter.
2. Prepare all media for growing yeast: YPG plates, YPD 4% plates, YPD, rYPD, SPO, and CuSO_4_.
3. Patch yeast strains stored at −80 °C onto a YPG plate and allow to grow at 30 °C for 16–18 h.

***Note:*** By growing cells on YPG, a non-fermentable carbon source, it is ensured that cells can undergo cellular respiration, a requirement for meiosis.

4. Transfer a toothpick of cells to a YPD 4% plate. Allow to grow at 30 °C for 24 h.
5. Inoculate a toothpick of cells into 40 mL of YPD liquid culture in a 500 mL Erlenmeyer flask. Shake at 30 °C for 6 h or until an OD_600_ reaches between 0.5 and 2.0.
6. Take the OD_600_ reading using a spectrophotometer and dilute cells to an OD_600_ of 0.05 in 100 mL rYPD. Grow in a 1 L Erlenmeyer flask for 16–18 h at 30 °C.

***Note:*** If performing with a different volume of cells, maintain a cell volume:flask capacity ratio of approximately 1:10 to ensure proper aeration.

### Installation of MinKNOW

**Timing: 1 h**

7. Upon purchase of a device from Oxford Nanopore Technologies (ONT), customers are provided access to their community site (https://community.nanoporetech.com/). Software downloads and protocols are accessible here. This site also hosts an active and useful forum to help with troubleshooting at every step from kit selection to analysis. Before performing a sequencing run with a MinION device, MinKNOW software must be installed on the computer that the MinION device will be connected to during sequence acquisition. To perform base calling simultaneously with sequence acquisition, the host computer must meet minimum host computer specifications. See the “Minimum host computer specifications document” at the community site for more information. If a host computer meeting the required specifications is not available, base calling can be performed after the fact using Guppy.

## Key resources table

**Table:** 

REAGENT or RESOURCE	SOURCE	IDENTIFIER
**Chemicals, peptides, and recombinant proteins**		

Acid Phenol:Chloroform	Invitrogen	AM9722
Adenine	Sigma-Aldrich	A8626-25g
Bacto^TM^ Dehydrated Agar	BD Difco^TM^	Cat#214010
Bacto^TM^ Peptone	BD Difco^TM^	Cat#211677
Bacto^TM^ Yeast Extract	BD Difco^TM^	Cat#212720
Cap-Clip acid pyrophosphatase	Tebu-Bio	C-CC15011H
Copper Sulfate	Sigma-Aldrich	451657-10g
Costar SpinX column	Corning Incorporated	CLS8161
Dextrose	Fisher Scientific	D1610
EB	QIAGEN	Cat#19086
EDTA (0.5 M)	Ambion	AM9260G
Glycerol	Fisher Scientific	G334
Glycogen	Invitrogen	AM9510
HighPrep PCR beads	MagBio	AC-60050
Histidine	Sigma-Aldrich	H5659-100g
KAPA Hi-Fi hot start ready mix	Roche	KK2601
KAPA single indexed adapters Set B	Roche	KK8702
Leucine	Sigma-Aldrich	L800-100g
Linear acrylamide	Ambion	AM9520
Low Molecular Weight DNA Ladder	New England BioLabs	N3233
MyOne Streptavidin C1 Dynabeads	Thermo Fisher Scientific	Cat#65001
Novex 6% TBE gels	Invitrogen	EC62655BOX
Recombinant shrimp alkaline phosphatase	New England BioLabs	M0371
R9.4.1 flow cell	Oxford Nanopore Technologies	FLO-MIN106
Raffinose	Sigma-Aldrich	R0250-500g
RNase cocktail enzyme mix	Ambion	AM2286
RNase H	New England BioLabs	M0297
RNasin Plus	Promega	N2611
SDS, 20% Solution, RNase-Free	Ambion	AM9820
Sodium Acetate (3 M)	Ambion	AM9740
Sodium Chloride (5 M)	Ambion	AM9759
SuperScript IV reverse transcriptase	Invitrogen	Cat#18090010
T4 RNA ligase 1	New England BioLabs	M0437M
Tris-HCl pH 7.5 (1 M)	Invitrogen	Cat#15567027
Tryptophan	Sigma-Aldrich	T0254-100g
Uracil	Sigma-Aldrich	U0750-25mg
RNA Fragmentation Reagent	Ambion	AM8740

**Critical commercial assays**		

Poly(A)Purist MAG kit	Ambion	AM1922
KAPA HyperPrep Kit	Roche	KK8504
Direct RNA Sequencing Kit	Oxford Nanopore Technologies	SQK-RNA002

**Deposited data**		

Sequencing from Tresenrider et al. (2021)	NCBI	GEO: GSE140177

**Experimental models: organisms/strains**		

*Species: S. cerevisiaeStrain: SK1Sex: MATa/MATalphaGenotype: pCUP1-IME1::NAT/pCUP1-IME1::NATpCUP1-IME4::NAT/pCUP1-IME4::NAT amn1(BY4741 allele) unmarked/amn1(BY4741 allele) unmarked*	Tresenrider et al. (2021)	N/A

**Oligonucleotides**		

5oligocap	Pelechano et al. (2013)	dCdAdCdTdCdTrGrArGrCrArArUrArCrC
Second strand biotinylated oligo	Wu et al. (2018)	GCAC/iBiodT/GCACTCTGAGCAATACC

**Software and algorithms**		

cutadapt, v2.3	Martin (2011)	https://cutadapt.readthedocs.io/en/stable/
STAR, v2.5.3a	Dobin et al. (2013)	https://github.com/alexdobin/STAR
BSgenome, v1.50.0	Pagè (2018)	https://bioconductor.org/packages/release/bioc/html/BSgenome.html
CAGEr, v1.24.0	Haberle et al. (2015)	https://bioconductor.org/packages/release/bioc/html/CAGEr.html
DESeq2, v1.22.2	Love et al. (2014)	https://bioconductor.org/packages/release/bioc/html/DESeq2.html
MinKNOW, v1.10.23	Oxford Nanopore Technologies	https://nanoporetech.com/
Guppy, v5.0.11	Oxford Nanopore Technologies	https://nanoporetech.com/
minimap2, v2.9-r720	Li (2018)	https://github.com/lh3/minimap2
Integrated Genomics Viewer	Robinson et al. (2011)	https://software.broadinstitute.org/software/igv/
custom code	Tresenrider et al. (2021)	https://github.com/atresen/LUTI_key_features

**Other**		

1 cm glass pre-filters	Whatman	Cat#1823010
18 G BD™ Needle 1 1/2 in. single use, sterile	BD	Cat#305196
2100 Bioanalyzer System	Agilent Technologies	G2939BA
47 mm filters (0.45 um pore)	Whatman	Cat#7184-004
KIMBLE ULTRA-WARE 47 mm Microfiltration Assembly	DWK Life Sciences	Cat#953780-0000
MinION	Oxford Nanopore Technologies	MIN-101B
NanoDrop 2000 Spectrophotometer	Thermo Fisher Scientific	ND-2000
Qubit 4 Fluorometer	Invitrogen	Q33238
Ultrospec 2100 pro	Amersham Biosciences	Cat#80-2112-21
DynaMag-2 Magnet	Invitrogen	12321D
DynaMag-PCR Magnet	Invitrogen	Cat#492025

## Materials and equipment

***Alternatives:*** In this protocol, a Bioanalyzer was used for library quality control at various steps. A TapeStation can be used in its place. They both provide information on the size and concentration of fragments in a sample. If neither machine is available, an agarose gel can be run to determine the approximate size of nucleotide fragments in the sample. The gel will not be quantitative.

***Alternatives:*** At all steps in which RNA or cDNA was quantified, Qubit reagents were used to determine the concentration. However, a NanoDrop is sensitive enough to quantify total RNA and/or polyA RNA abundance in steps 23 and 31. When performing quantification of cDNA, the Qubit should be used because a NanoDrop is not sensitive enough to accurately quantify the low cDNA concentration of the library.

***Alternatives:*** If Shrimp Alkaline Phosphatase (rSAP) is not available, Antarctic Phosphatase is an ideal alternative. Both are preferred over Calf Intestinal Alkaline Phosphatase as they are more amenable to rapid and complete heat inactivation.

***Alternatives:*** For mRNA selection, the Poly(A)Purist MAG kit was used. However, any poly(A) purification kit that enables sufficient removal of rRNA and recovery of mRNA can be substituted. rRNA depletion methods could also be used to enrich for mRNAs; however, when using these methods, even after depletion, many non-mRNA RNA species will remain in the sample. Because the protocol described below specifically targets capped mRNA sequences, any additional RNA species present after rRNA depletion will not be captured for sequencing.

***Alternatives:*** We used Qiagen RNeasy Mini Columns to perform RNA size selection after fragmentation and to remove adaptors after ligation. It is possible to use a bead-based method instead. The MAGBIO HighPrep RNA Elite beads are one option. If using beads, it is critical that they are 1) designed for RNA and 2) are RNase free.

***Alternatives:*** For library preparation, the KAPA HyperPrep Kit can be substituted with any library prep kit that prepares Illumina sequencing compatible libraries from fragmented dsDNA.

### Cell culture media and solutions

**Table:** 4 % Agar

Reagent	Final concentration	Amount
Agar	4 % (w/v)	10 g
ddH_2_O	n/a	250 mL
**Total**	**n/a**	**250 mL**

Autoclave. The agar will dissolve into solution during the autoclaving process. Store at 4 °C for up to 3 months.

**Table:** YPG plates

Reagent	Final concentration	Amount
Agar (4 % (w/v))	2 % (w/v)	250 mL
Yeast Extract	1 % (w/v)	5 g
Peptone	2 % (w/v)	10 g
Glycerol (30 % (v/v))	3 % (v/v)	50 mL
ddH_2_O	n/a	See note below
**Total**	**n/a**	**500 mL**

Mix everything except the agar together, bring to a volume of 250 mL with ddH_2_O, and filter sterilize. Separately, melt the solidified 4 % agar in a microwave until just dissolved. Mix in the melted agar and pour 25 mL per plate. Store the plates at 4 °C for up to 6 weeks.

**Table:** YPD 4 % plates

Reagent	Final concentration	Amount
Agar (4 % (w/v))	2 % (w/v)	250 mL
Yeast Extract	1 % (w/v)	5 g
Peptone	2 % (w/v)	10 g
Dextrose (40 % (w/v))	4 % (w/v)	50 mL
ddH_2_O	n/a	See note below
**Total**	**n/a**	**500 mL**

Mix everything except the agar together, bring to a volume of 250 mL with ddH_2_O, and filter sterilize. Separately, melt the solidified 4 % agar in a microwave until just dissolved. Mix in the melted agar and pour 25 mL per plate. Store the plates at 4 °C for up to 6 weeks.

**Table:** YPD

Reagent	Final concentration	Amount
Yeast Extract	1 % (w/v)	10 g
Peptone	2 % (w/v)	20 g
Dextrose (20 % (w/v))	2 % (w/v)	100 mL
Tryptophan	9.6 mg/L	9.6 mg
Uracil	2.4 mg/L	2.4 mg
Adenine	1.2 mg/L	1.2 mg
ddH_2_O	n/a	to 1 L
**Total**	**n/a**	**1 L**

Filter sterilize and store at 20 °C–25 °C for up to 3 months. Discard if the liquid becomes cloudy or particulates are observed.

**Table:** rYPD

Reagent	Final concentration	Amount
Yeast Extract	1 % (w/v)	10 g
Peptone	2 % (w/v)	20 g
Dextrose (20 % (w/v))	1 % (w/v)	50 mL
Uracil	2.4 mg/L	2.4 mg
Adenine	1.2 mg/L	1.2 mg
ddH_2_O	n/a	to 1 L
**Total**	**n/a**	**1 L**

Filter sterilize and store at 20 °C–25 °C for up to 3 months. Discard if the liquid becomes cloudy or particulates are observed.

**Table:** SPO

Reagent	Final concentration	Amount
Potassium Acetate	1 % (w/v)	10 g
Adenine	40 mg/L	40 mg
Uracil	40 mg/L	40 mg
Histidine	20 mg/L	20 mg
Leucine	20 mg/L	20 mg
Tryptophan	20 mg/L	20 mg
Raffinose (20 % (w/v))	0.02 % (w/v)	1 mL
ddH_2_O	n/a	to 1 L
**Total**	**n/a**	**1 L**

pH to 7.0 with glacial acetic acid, filter sterilize, and store at 20 °C–25 °C for up to 3 months.

**Table:** CuSO_4_

Reagent	Final concentration	Amount
CuSO_4_	100 mM	15.96 mg
ddH_2_O	n/a	1 mL
**Total**	**n/a**	**1 mL**

Store at 20 °C–25 °C for up to 1 year.

### Solutions for RNA extraction and TL-seq

**Table:** TES

Reagent	Final concentration	Amount
Tris-HCl pH 7.5 (1 M)	10 mM	5 mL
EDTA (0.5 M)	10 mM	10 mL
SDS (20 % (w/v))	0.5 % (w/v)	12.5 mL
ddH_2_O	n/a	472.5 mL
**Total**	**n/a**	**500 mL**

Filter sterilize and store at 20 °C–25 °C for up to 2 years.

**Table:** Crush and Soak buffer

Reagent	Final concentration	Amount
Sodium Chloride (5 M)	500 mM	10 mL
EDTA (0.5M)	1.0 mM	200 μL
SDS (20 % (w/v))	0.05 % (w/v)	250 μL
ddH_2_O	n/a	89.55 mL
**Total**	**n/a**	**100 mL**

Filter sterilize and store at 20 °C–25 °C for up to 2 years.

## Step-by-step method details

### Preparation for cell filtration

**Timing: 10 min (on the day of the experiment)**

The filtration apparatus which will be used to filter cells in step 10 is setup.

1. Prepare a Whatman filter funnel on top of an Erlenmeyer filter flask (Figure 1).

**Figure 1:**
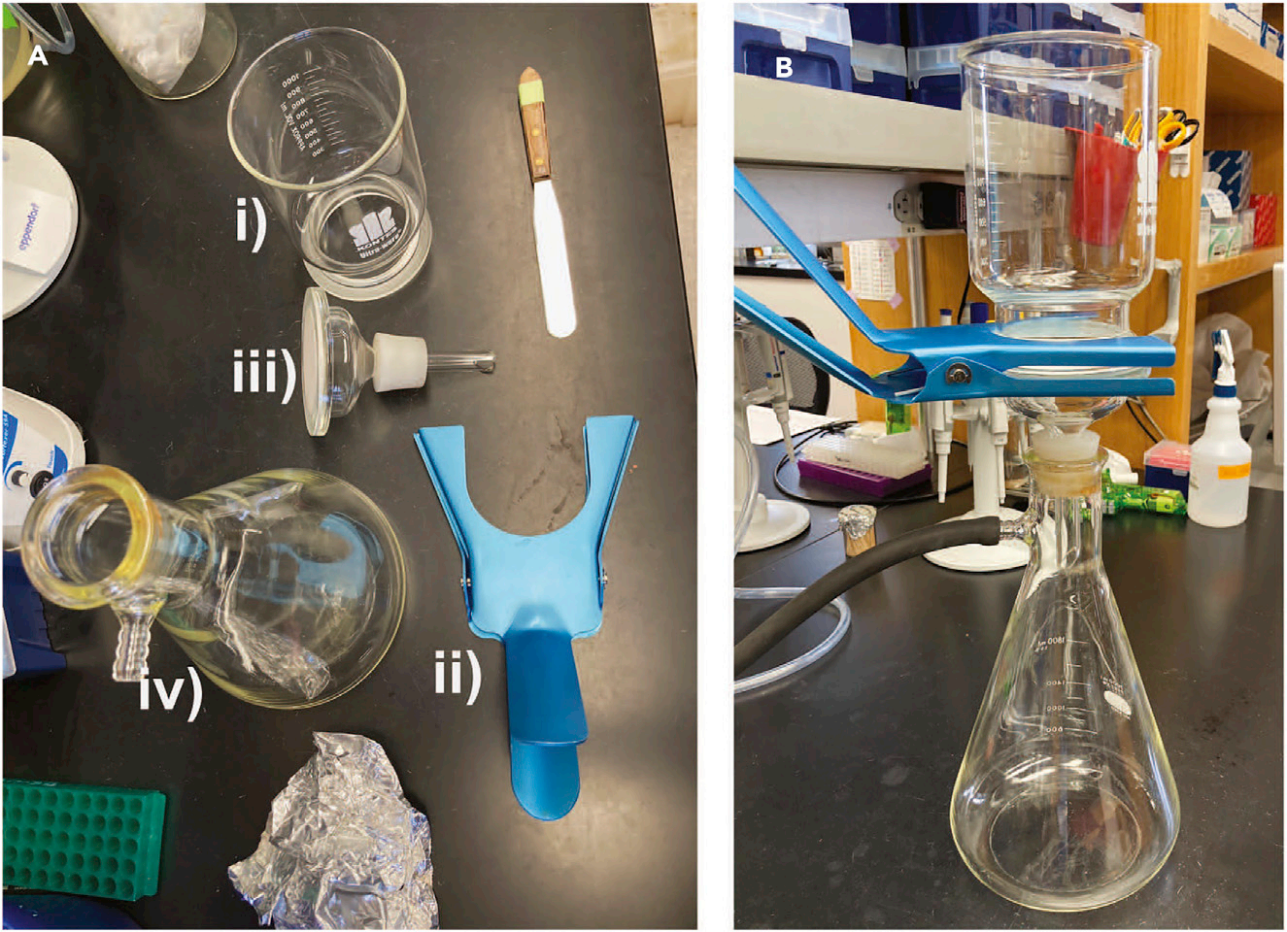
Preparation of a filtering apparatus (A) The parts of the apparatus are labeled i–iv from top to bottom. (B) An assembled apparatus. The reservoir (**i**) is placed on top of the filter (**iii**). In between **i** and **iii** is a piece of 47 mm (0.45 μm pore) filter paper. These two pieces are held together by a clamp (**ii**). This assembled piece is then placed upon a filtration flask (**iv**) which is attached to a vacuum.
2. Before adding cells to the filter, turn on the vacuum and wet the filter paper completely with deionized water.
3. Fill a clean container with liquid nitrogen.
4. For each sample to be collected, puncture the lid of a 50 mL conical tube using an 18G needle. This hole allows for the evaporation of liquid nitrogen.
5. Fill the conical with liquid nitrogen to 1/3 full and place it into the clean container filled with liquid nitrogen.

### Meiotic induction and cell collection

**Timing: 5 h**

Cells are induced to undergo meiosis and then harvested at the indicated timepoints.

***Note:*** A minimum of two, but ideally three or more replicates should be performed. Collect replicates on separate days.

***Note:*** We wanted to minimize added stressors to cells, so we opted to collect the cells by filtration immediately followed by cryopreservation in liquid nitrogen. Alternatives, such as collection by centrifuge, were not tested.

6. Take the OD_600_ for cells grown for 16 h in rYPD and calculate the volume of cells required to reach 250 OD_600_ units. Pipet that volume into a 50 mL conical tube.

***Note:*** Do not use the 50 mL conical in liquid nitrogen.

***Note:*** Aim for OD_600_ > 6 for optimal meiotic synchrony and efficiency.

7. Centrifuge at 2000 g for 2 min at 25 °C, pour off the liquid, and resuspend in 40 mL milliQ water.
8. Centrifuge at 2000 g for 2 min at 25 °C, pour off the liquid, and resuspend in SPO to a final volume of 100 mL in a 1000 mL Erlenmeyer flask.
9. Shake at 300 rpm at 30 °C for 2 h.

***Note:*** By removing a fermentable carbon source from the cultures, the cells switch to cellular respiration. This primes the cells for entry to meiosis; however, because the meiotic regulators *IME1* and *IME4* are controlled by the *CUP1* promoter, they will not enter the meiotic program until exposed to CuSO_4_.

**Figure 2:**
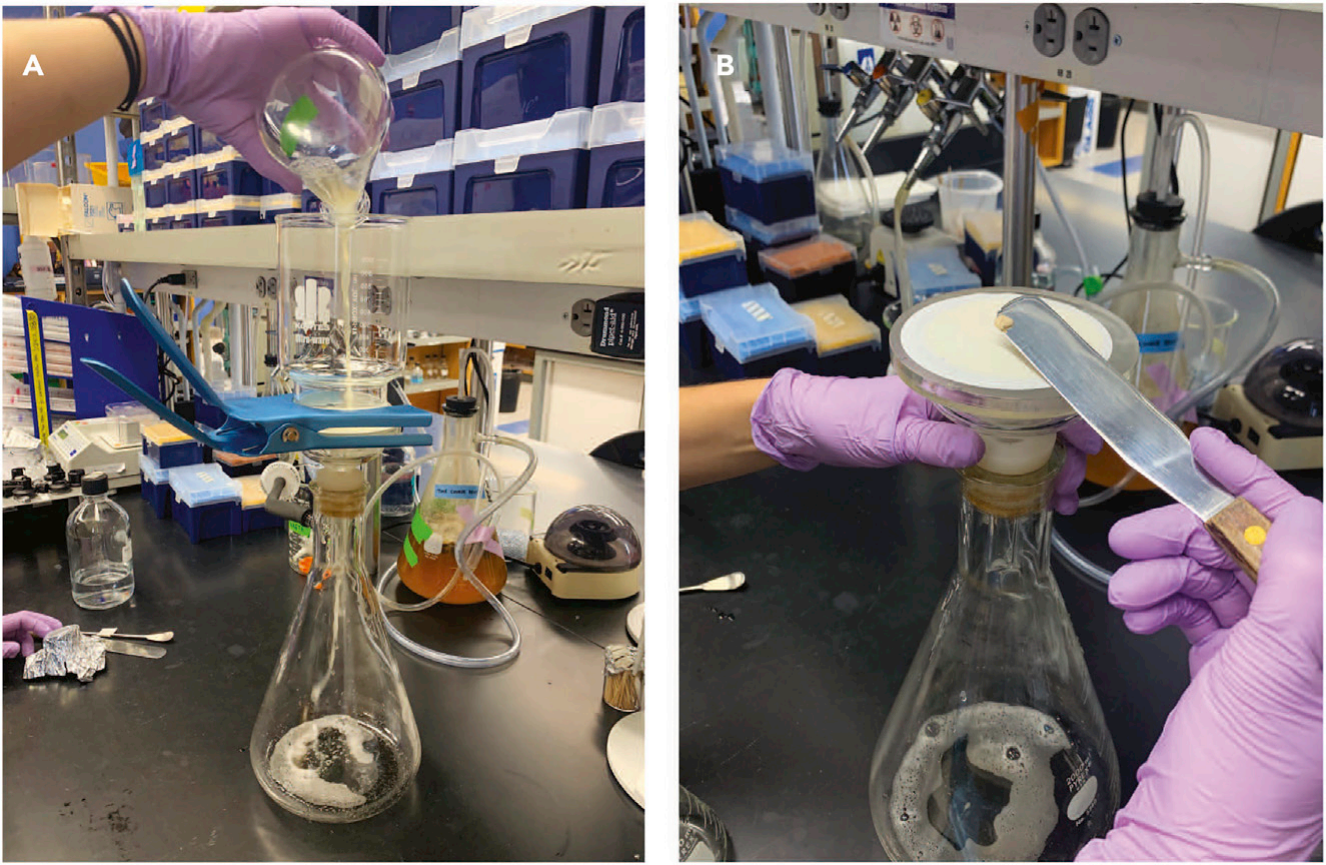
Cell filtration and harvesting (A) With the vacuum on, pour the cells into the reservoir. Allow the liquid to be vacuumed into the flask below. (B) When no liquid remains in the reservoir, remove the clamp and expose the filter paper with cells on it. Using a sterilized spatula, scrape the cells from the filter paper.
  b. Remove the clamp and funnel from the filtration apparatus and scrape up the cells using an ethanol sterilized spatula. Allow the cells to build up on the spatula (Figure 2B).
  c. Place the spatula with cells into the clean container filled with liquid nitrogen. Keep the cells submerged for 20 s to snap freeze them.
  d. Dip the spatula with the cells into the 50 mL conical 1/3 full of liquid nitrogen.
  e. Lay the punctured cap onto the conical. At this point the spatula is still in the tube, so the cap will not sit flush on the conical. While pressing on the cap, pull the spatula from the tube in a manner that breaks the cell pellet from the spatula, leaving the frozen cells in the conical.
    ***Note:*** The cells will be stuck in a hard frozen clump to the spatula. Pressure between the rim of the conical tube and its cap is used to break the hard mass of frozen cells away from the metal spatula.
  f. There will be 100 OD_600_ units of cells in the tube.
    ***Note:*** This is a large number of cells, but it is the minimum that we have tested. The TL-seq method to be described below requires a high cell input due to multiple rounds of processing and the selection of only the most 5′ end of capped transcripts.
  g. Screw the cap onto the tube before storing at −80 °C.
    **Pause point:** The frozen cells can be stored at −80 °C for at least 3 months.

10. Collect 40 mL of cells by filtration.
  a. Pour the cells into the funnel attached on top of the filtration apparatus and filter out the liquid via vacuum (Figure 2A).

11. To the remaining 60 mL of cells, add 30 μL of 100 mM CuSO_4_ and continue shaking at 30 °C for 2 h.
  ***Note:*** This initiates meiosis by inducing expression of *IME1* and *IME4.*
12. At 2 h after addition of CuSO_4_ and 4 h after transfer to SPO, collect the second timepoint (see step 10).
13. Transfer the remaining 20 mL of cells to a new 250 mL Erlenmeyer flask and continue shaking at 30 °C until > 24 h after transfer of the cells to SPO.
14. Under a brightfield microscope, determine the sporulation status of at least 100 cells to calculate the sporulation efficiency.

**CRITICAL:** At least 90 % of cells should have completed sporulation. See problem 1 in the Troubleshooting section for additional assistance.

### Total RNA extraction and poly(A)-selection

**Timing: 3 days**

Cells are lysed and a total RNA extraction is performed. Poly(A) selection isolates mRNA which can then be used for direct RNA sequencing or TL-seq.

15. Thaw 100 OD_600_ units of cells per time point on ice.
16. Add 5 mL of TES, vortex to resuspend, and aliquot 10 × 500 μL into 1.5 mL screw cap tubes for each timepoint (20 tubes total for 2 timepoints).

***Note:*** For every 10 OD_600_ units of cells, resuspend in 500 μL of TES. If greater than 100 OD_600_ units of cells are collected, increase the total volume of TES proportionally. Keep the volume of TES per tube constant (500 μL) by increasing the number of tubes used.

17. Add 500 μL of acid phenol to each tube. Incubate at 65 °C for 45 min in a Thermomixer C shaking at 1400 RPM. Centrifuge at room temperature for 5 min at 20,000 *g*.
18. Transfer the aqueous phase to 1 mL of cold 100 % ethanol and 40 μL of 3 M sodium acetate using a P200 pipet. Invert to mix, and incubate > 16 h at −20 °C.

**Pause point:** RNA can be stored at this point for at least 2 weeks.

19. Retrieve samples and centrifuge the samples at 20,000 *g* for 30 min at 4 °C. Carefully aspirate the liquid and wash in 1 mL of 80 % ethanol.
20. Centrifuge at 20,000 g for 5 min to bring the RNA pellet to the bottom of the tube. Carefully aspirate the 80 % ethanol until 100 μL remains.
21. Perform an additional short spin and remove residual ethanol with a P200 pipet. Dry pellets in the hood for 25 min or until dry.
22. Resuspend in 30 μL of nuclease-free water by incubating in a Thermomixer at 37 °C with shaking (1400 RPM).
23. Combine all tubes for each timepoint, quantify total RNA using the Qubit RNA BR Assay Kit, and check RIN score using a Bioanalyzer with an RNA Analysis kit.

***Note:*** From an input of 100 OD_600_ units of cells, expect 1–2 mg of total RNA.

24. To 1 mg total RNA, spike in 25 ng of pooled *in vitro* transcripts (IVTs). IVTs can be prepared as described in Box 1 of Pelechano et al. (2014). These are transcripts added in known amounts that can be used for quality control during analysis.
25. Add nuclease-free water to bring the RNA concentration to 600 μg/mL in a 2 mL tube. The final volume should be 833 μL per timepoint.
26. Follow the Poly(A)Purist MAG kit instructions to isolate poly(A) specific RNA.

***Note:*** There are many commercial poly(A) selection kits that could be used in place of the Poly(A)Purist MAG kit. Most critical is that 5–10 μg of mRNA are isolated and rRNA cannot be seen on Bioanalyzer traces after completion of the poly(A) selection.

27. Premix 40 μL 3 M Sodium acetate, 1 μL glycogen (5 mg/mL) and 1.1 mL ethanol in a fresh tube. Add the eluted poly(A) RNA mixture and mix by inversion. Leave the RNA to precipitate at –20 °C for > 16 h or at –80 °C for 1 h.

**Pause point:** RNA can be stored up to a month at this point.

28. Recover the RNA by centrifugation at ≥12,000 *g* for 30 min at 4 °C. Carefully remove and discard the supernatant. RNA pellet might be loose.
29. Add 1 mL 80 % ethanol and vortex the tube a few times. Pellet the RNA by centrifuging for 10 min at 4 °C. Remove supernatant.
30. Dissolve the poly(A) RNA pellet in 21 μL pre-heated nuclease-free water (NO EDTA) by vortexing briefly for several seconds.

***Note:*** The nuclease-free water should be pre-heated to 60 °C–80 °C to help the RNA dissolve into solution.

**Pause point:** Flash freeze the RNA in liquid nitrogen and store at −80 °C if not continuing with the protocol immediately. The RNA can be stored at −80 °C for several months.

31. Quantify the extracted RNA using Qubit RNA BR reagents.

***Note:*** A return of 1 % of input material is expected during poly(A) selection. With an input of 1 mg of RNA, between 5 and 15 μg of poly(A) selected RNA is an ideal recovery range.

### Nanopore direct RNA sequencing and analysis

**Timing: Library prep and loading 3 h, data acquisition and processing up to 3 days**

Quality checked poly(A) selected mRNA is prepared for sequencing and loaded onto ONT’s MinION device. The output data is processed, aligned to the SK1 *Saccharomyces cerevisiae* genome, and the sequenced transcripts are visualized.

32. Before proceeding with preparation of a library for direct RNA sequencing, check the quality of the poly(A) selected RNA.
  a. Assess fragment length using a Bioanalyzer with an RNA Analysis kit.
    i. Ideal average fragment size 2000 bp
  b. Check the RNA purity by Nanodrop.
    i. Ideal A260:A280 ratio of 2.0
    ii. Ideal A260:A230 ratio of 2.0–2.2
33. Dilute 500 ng of poly(A) selected RNA to 9 μL in nuclease-free water.
34. Continue following the “Direct RNA sequencing” protocol from ONT for use with the kit (SQK-RNA002)
35. To setup and perform the sequencing run, refer to ONT’s “MinKNOW Protocol”.

**CRITICAL:** If the host computer does not meet the minimum specifications, live base calling should be turned off. Base calling can be performed later with a compatible computer using the standalone Guppy base calling software.

***Note:*** The Albacore software was used for base calling in Tresenrider et al., (2021). This program is no longer supported and has been replaced by Guppy.

***Optional:*** If base calling was not performed live, run guppy_basecaller in which --input_pathpoints towards the directory containing the fast5 files outputted by MinKNOW.

### Transcript leader sequencing (TL-seq) library preparation

**Timing: 5 days**

Quality checked poly(A) selected mRNA is used in the construction of TL-seq libraries.

**Figure 3:**
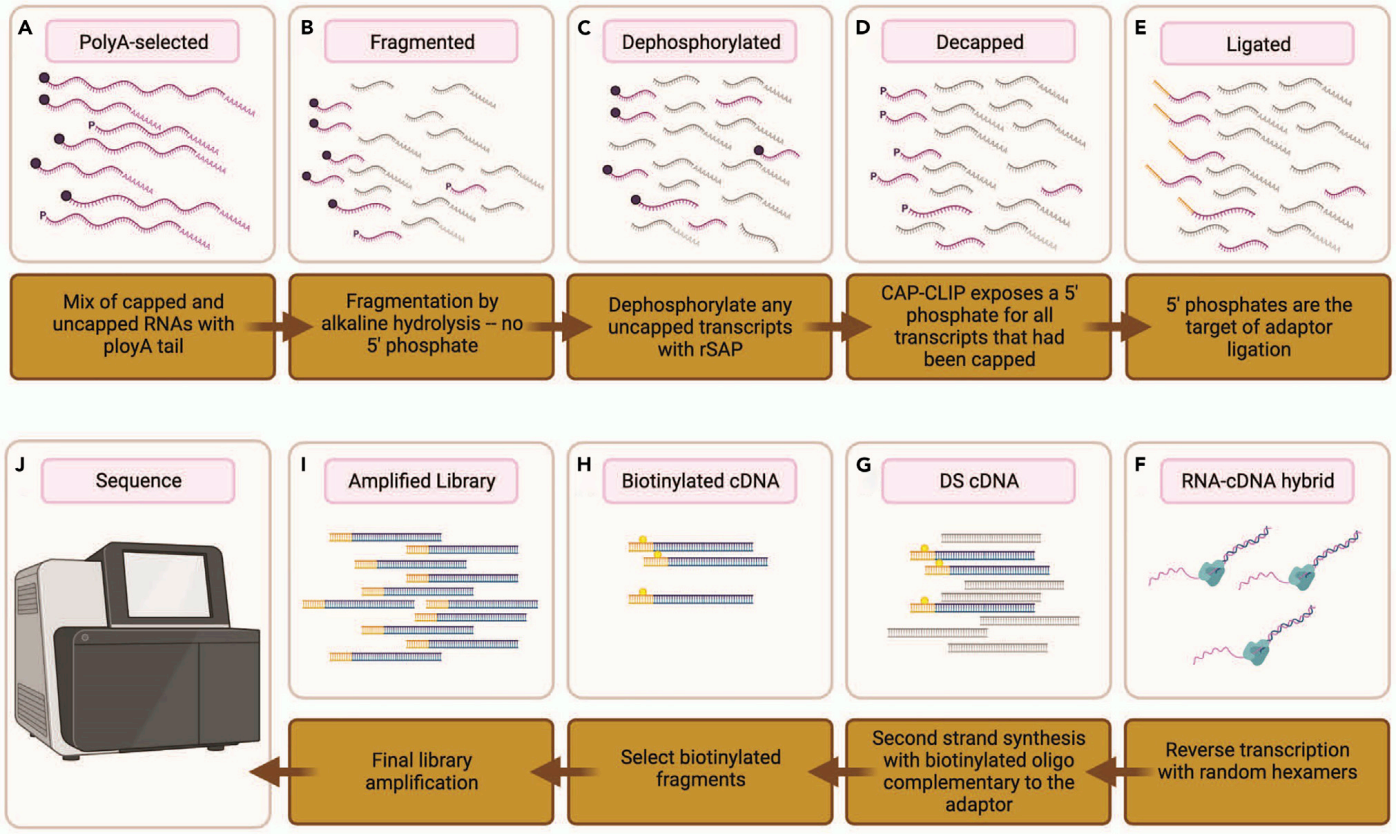
An illustration of key steps in the TL-seq protocol (A-J).

***Note:*** This protocol is adapted from Wu et al. (2018) and was also used in Chia et al. (2021). Figure 3 provides an overview of the critical steps in TL-seq.

36. Use the remaining poly(A) selected RNA for TL-seq library preparation (Figure 3A).
37. Follow the instructions for the RNA Fragmentation Reagent (Ambion) to fragment the RNA (Figure 3B).

**CRITICAL:** In our experience, 3 minutes fragmented the RNA from meiotic yeast cells into 200 bp fragments.

**CRITICAL:** Alkaline hydrolysis at 70 °C is performed to shear the RNAs into uniform length fragments. Depending on the average physical length of the transcriptome, different fragmentation times may be needed to fragment RNAs in a population to a specific size. Hence, the best fragmentation time must be determined in a test experiment which is recommended to be performed before any larger experiment with multiple samples. In order to test the hydrolysis at multiple times, the cell collection should be scaled up to produce 5 μg of poly(A) selected RNA for each of the times to be tested.

***Note:*** This fragmentation method leaves a 5′-OH and a 3′-phosphate which prevents RNA-ligation onto the fragment by T4 RNA Ligase. This necessitates downstream end-repair with rSAP or equivalent enzymes.

38. Use a Bioanalyzer with RNA reagents to determine the average size of the fragmented RNA.

***Note:*** See Expected Outcomes for an example of the RNA fragments before and after fragmentation.

39. Size select 200–300 bp fragments using a Qiagen RNeasy Mini Kit following the manufacturer’s instructions for RNA Cleanup. Elute the RNA from the column in 14 μL of nuclease-free water. Perform a second elution in 14 μL of nuclease-free water such that the final eluted volume will be 24 μL.
40. Check the fragment size by Bioanalyzer with RNA reagents.
41. To remove 5′-phosphates from uncapped fragments (Figure 3C), setup the reaction below in a 1.5 mL tube.
  **Table:** Phosphatase reaction mix

Reagent	Amount
RNA sample (up to 5 μg)	10 μL
NEB CutSmart buffer (10×)	20 μL
Shrimp Phosphatase (rSAP) (1 U/μL)	20 μL
RNasin PLUS	2 μL
nuclease-free water	148 μL

  **CRITICAL:** Because we aim to sequence only the most 5′end of transcripts, we do not want to prepare a library dominated by fragments from uncapped transcripts. Dephosphorylation of the 5′-end of uncapped fragments will prevent the ligation of adaptors to these RNA fragments in future steps.
  ***Note:*** Shrimp Alkaline Phosphatase (rSAP) also repairs the 3′-ends of alkaline fragmented RNA to 3′-OH making them ligation competent.
  a. Incubate the samples for 1 h at 37 °C.
  b. Heat inactivate at 65 °C for 5 min.
  c. Add 200 μL acid phenol and incubate at 65 °C for 45 min in a Thermomixer C shaking at 1400 RPM.
  d. Centrifuge at room temperature for 5 min at 20,000 *g*.
  e. Using a P200 pipet, transfer the aqueous phase to 1 mL of cold 100 % ethanol, 40 μL of 3 M sodium acetate, and 1 μL of linear acrylamide.
  f. Invert to mix and incubate > 16 h at −20 °C or 1 h at −80 °C.
    **Pause point:** RNA can be stored at this point for at least one month.
  g. Retrieve samples and centrifuge the samples at 20,000 *g* for 30 min at 4 °C. Carefully aspirate the liquid and wash in 1 mL of 80 % ethanol.
  h. Centrifuge at 20,000 *g* for 5 min to bring the RNA pellet to the bottom of the tube. Carefully aspirate the 80 % ethanol until 100 μL remains.
  i. Perform an additional short spin and remove any residual ethanol with a P200 pipet. Dry pellets in the hood.
  j. Resuspend in 13 μL of nuclease-free water.

42. In each tube, setup the reaction below to de-cap the capped transcripts (Figure 3D).
  **CRITICAL:** Because the dephosphorylation reaction does not remove 100 % of 5′-phosphates, some will remain. These fragments should be considered noise as they do not represent the true 5′-end of a transcript. It is important to sequence at least one library that includes only the signal from these background fragments. This can be done by splitting the sample into two before the de-capping reaction and treating only one half of the RNA with the CAP-CLIP enzyme, which removes the m7G cap from mRNA fragments. All subsequent steps should be applied to both the + and – CAP-CLIP reactions. We recommend performing this control for one sample per timepoint if processing all replicates at the same time.
  **Table:** CAP-CLIP reaction mix

Reagent	Amount
RNA sample diluted with water	13 μL
CAP-CLIP reaction buffer (10×)	2 μL
DTT (10 mM)	2 μL
CAP-CLIP (10 U/μL)	2 μL
RNasin PLUS	1 μL

  a. Incubate the samples for 1 h at 37 °C.
  b. Perform an acid phenol-based ethanol precipitation as in the previous step.
  c. Resuspend in 12 μL nuclease-free water.

43. Setup the ligation reaction below in a PCR tube (Figure 3E).
  **Table:** 5′-adaptor ligation reaction mix

Reagent	Amount
RNA sample	12 μL
5oligocap (100 μM)	2 μL
Buffer T4 RNA ligase I	2 μL
10 mM ATP	2 μL
T4 RNA ligase 1, (30,000 units/mL)	1 μL
RNasin Plus	1 μL

  a. Incubate at 16 °C for 16 h in a thermocycler with the heated lid turned off.
  b. Purify the ligation on a Qiagen RNeasy Mini column to remove excess un-ligated adapter.
  c. Elute in 14 μL, the final eluted volume will be 11 μL due to volume loss from the column purification.

44. Setup the RNA denaturation reaction below in a PCR tube.
  **Table:** RNA denaturation reaction mix

Reagent	Amount
RNA sample (<5 μg RNA)	X μL
Random hexamers (50 μM)	1 μL
RNasin	1 μL
10 mM dNTP mix (each)	1 μL
Water	(11 μL – X μL)

  a. Denature at 65 °C for 5 min. Immediately place on ice for at least 1 min.
  b. During this step, prewarm 5× SSIV buffer at room temperature. Vortex to mix well and then centrifuge briefly.

45. To each tube, add 6 μL of the mixture below to bring the total volume (step 44 + 45) to 20 μL.

**Table:** RT reaction mix

Reagent	Amount
5X SSIV buffer	4 μL
100 mM DTT	1 μL
SSIV enzyme	1 μL

46. Incubate in a thermocycler with the following conditions to complete the reverse transcription (Figure 3F)

**Table:** Incubation conditions

Steps	Temperature	Time	Cycles
Annealing	23 °C	10 min	1
Reverse Transcription	50 °C	10 min	1
Heat Inactivation	80 °C	10 min	1
Hold	4 °C	forever	1

47. Degrade the template RNA with 1 μL of the mixture below and incubate at 37 °C for 30 min.

**Table:** RNA degradation reaction mix

Reagent	Amount
RNase H (5 U/μL)	0.5 μL
RNase cocktail	0.5 μL

48. Purify the sample using 1.8× HighPrep PCR Clean-up beads (37.8 μL beads for a sample volume of 21 μL) according to the manufacturer’s instructions.
  a. Elute the sample in 23.5 μL of nuclease-free water.

49. Set up the following 50 μL reaction ON ICE using an internally biotinylated primer (Figure 3G).
  ***Note:*** Add the KAPA Hi-Fi hot start ready mix 2× last and on ice due to strong 3′–5′ exonuclease activity
  **Table:** Second strand cDNA synthesis reaction mix

Reagent	Amount
cDNA template (less than 1 μg)	23.5 μL
Second strand biotinylated oligo (10 μM)	1.5 μL
KAPA Hi-Fi hot start ready mix 2×	25 μL

  a. Use the following thermocycler settings for second strand cDNA synthesis (adapted from Adjalley et al., 2016)
    **Table:** Incubation Conditions

Steps	Temperature	Time	Cycles
Initial Denaturation	95 °C	3 min	1
Denaturation	98 °C	15 s	1
Annealing	50 °C	2 min	1
Extension	65 °C	15 min	1
Hold	4 °C	forever	1

50. Perform a 1.8× MagBio bead cleanup (90 μL beads for a sample volume of 50 μL).
  a. Elute in 21 μL of nuclease-free water
51. Measure the dsDNA using Qubit DNA HS reagents.

***Note:*** Expect to recover < 25 ng of adaptor-ligated cDNA.

52. Take up to 25 ng of sample and dilute to 50 μL with nuclease-free water.
53. Construct libraries using the KAPA HyperPrep kit with a few modifications from the manufacturer's instructions.
  a. After the Post-ligation Cleanup, resuspend the beads in 20 μL of nuclease-free water.
    **CRITICAL:** Before PCR amplification of the library, the biotinylated fragments need to be isolated. This step is NOT included in the KAPA HyperPrep kit instructions.
  b. While performing the Adapter Ligation and Cleanup, prepare Dynabeads MyOne Streptavidin T1 beads following the manufacturer’s instructions.
  c. Add 20 μL of washed Dynabeads MyOne Streptavidin T1 beads to 20 μL adapter-ligated cDNA and incubate for 30 min at 25 °C with rotation. Put on magnet for 2–3 min before removing supernatant (Figure 3H).
  d. Wash 1 time with 100 μL 1× B and W buffer. Do NOT re-suspend beads.
  e. Wash 1 time with Qiagen EB. Do NOT re-suspend beads.
  f. Re-suspend beads in 20 μL of milliQ water.
    **CRITICAL:** This is NOT an elution step. Samples are still bound to beads
  g. Take re-suspended beads and heat in a thermocycler at 90 °C for 5 min. Cool on ice.
  h. Continue with Library Amplification and Post Amplification Cleanup as described in the KAPA HyperPrep Kit instructions (Figure 3I).
  i. Re-suspend the beads in 20 μL EB.
  j. Incubate at RT for 2 min to elute DNA.
  k. Capture the beads with a magnet and transfer supernatant to a new tube.

54. Load 12.5 μL sample + 2.5 μL 6× purple gel loading dye onto 6 % TBE gels with 15 or 12 well combs (1.0 mm).

**CRITICAL:** Load at least one lane with Low MW DNA ladder (NEB).

55. Run at 120 V for 60 min or until the purple dye reaches the end of the gel.
56. Stain in SYBR Gold (in 1× TBE) for at least 1 h.
57. Visualize bands using a blue light transilluminator and excise DNA up to the range of 700 bp . Make sure to avoid including adapter-dimer bands that run close to the 150 bp band.

***Note:*** See expected outcomes for an example of what to expect and where to excise.

58. Prepare 1.5 mL tubes by removing the top of the tube and piercing 3 holes in the bottom using a 21G needle. Place a gel fragment inside and then place the tubes in a 2 mL collection tube. Spin 20,000 g for 3 min to shred gel.
59. Add 500 μL of Crush and Soak gel buffer. Incubate in a Thermomixer at 65 °C for 2 h (15 s shaking at 1000 rpm, 45 s rest).
60. Transfer the liquid portion of the supernatant into a Costar SpinX column into which two 1 cm glass pre-filters have been placed. Spin at 20,000 g for 1 min.

***Note:*** Gel chunks will remain in the column.

61. Add 1.5 mL EtOH + 60 μL 3M sodium acetate + 1 μL linear acrylamide to the flowthrough, precipitate for 16 h at −20 °C.
  a. Centrifuge for 30 min at 20,000 *g* and 4 °C.
  b. Wash the pellet with 80 % EtOH and centrifuge again for 10 min at 20,000 *g* and 4 °C.
  c. Remove the supernatant and re-suspend in 21 μL EB.
62. Quantify the cDNA by Qubit using HS reagents.

***Note:*** A successful library should have at least 25 ng–1 μg of final product.

63. Send for 75–100 bp single-end sequencing with 8 bp indices. Sequence to a depth of ∼20 million reads per sample.

## Expected outcomes

The final expected outcome of this protocol is the ability to confidently identify all 5′-extended transcripts during budding yeast meiosis. It should also be possible to quantify the transcripts being produced from both gene-distal promoters and the canonical gene-proximal promoters. To ensure the protocol is proceeding towards this intended goal, we provide here several expected outcomes at specific benchmarking steps.

**Figure 4:**
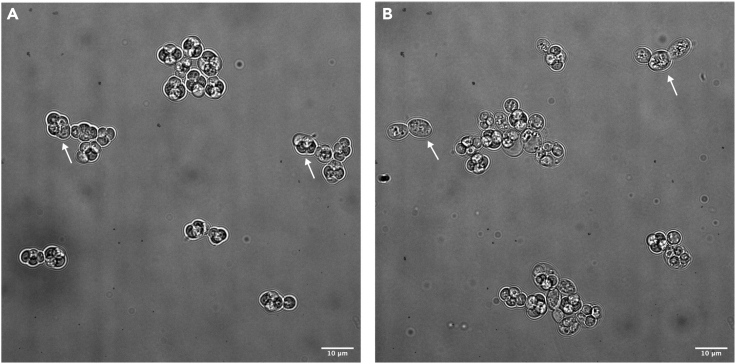
Sporulation efficiency (A and B) Example bright field images of cells that have undergone: (A) ideal meiosis (arrows indicate fully sporulated tetrads) or (B) poor meiosis (arrows indicate cells that have not sporulated). Scale bar is 10 μm.

First, it is critical to collect cells undergoing meiosis at a high efficiency. To determine the sporulation efficiency, the sporulation status of at least 100 cells should be counted using bright field microscopy after > 24 h in SPO (Figure 4). If fewer than 90 % of cells produce tetrads, review the recommendations in the troubleshooting section for tips to improve meiotic progression.

Second, it is crucial to extract and then process high quality RNA. In the table below, we outline the expected RNA/cDNA yields at critical points in the protocol. If the yield is lower than expected, refer to the troubleshooting section to determine possible causes and recommendations.

**Table:** Expected recovery

Input	Amount input	Output	Expected output
Whole meiotic cells	100 OD_600_ units	Total RNA	1–2 mg
Total RNA	1 mg	Poly(A) RNA	> 5 μg
poly(A) RNA	All extracted	cDNA	< 1 μg
cDNA (prior to selection of biotinylated fragments)	25 ng	Final Library	25 ng–1 μg

**Figure 5:**
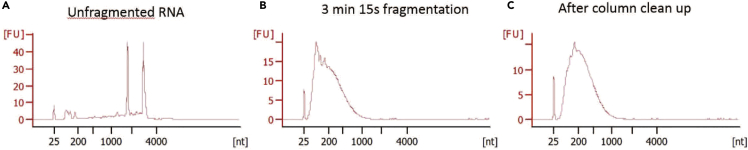
RNA before and after fragmentation (A) A bioanalyzer trace of total RNA. The two peaks are the 28S and 18S rRNAs. (B) A bioanalyzer trace of the poly(A) selected RNA after fragmentation by alkaline hydrolysis. (C) A bioanalyzer trace of the fragmented RNA after column purification. The fragment length is centered around 200 bp.

When performing RNA fragmentation, treatment time should be optimized to obtain fragments 200 base pairs long (Figure 5).

**Figure 6:**
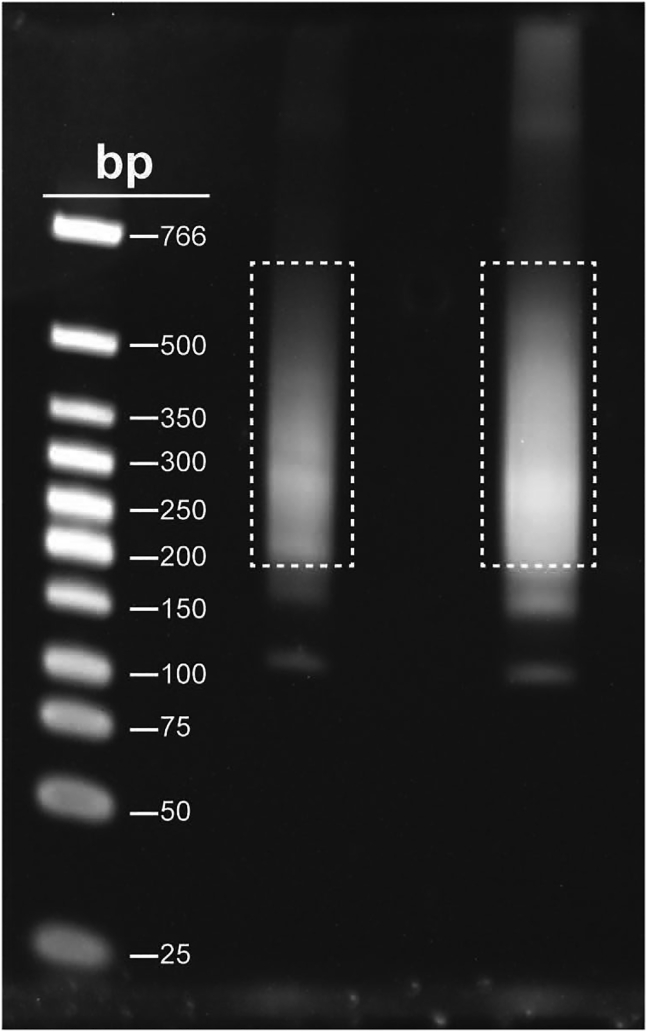
Gel extraction of final library After PCR, the final library is run on a 6 % TBE agarose gel. The boxes indicate the regions of the gel that were extracted (200–700 bp). Note that there is a band around 150 bp. Do not extract this as it includes adapter dimers.

Lastly, we provide examples of what a successful library should look like when run on a 6 % TBE gel prior to gel extraction (Figure 6).

## Quantification and statistical analysis

This step utilizes both TL-seq and direct RNA sequencing to identify full-length LUTI candidates and then quantify LUTI and PROX abundances.

1. Direct RNA libraries were sequenced on a MinION device as described above.
2. Fastq files outputted from Guppy were aligned with minimap2 (https://github.com/lh3/minimap2) using the SK1 genome assembled by combined PacBio and Illumina sequencing (Yue et al., 2017; https://yjx1217.github.io/Yeast_PacBio_2016/data/).
  $ minimap2 -ax splice -k14 -uf SK1.fa reads.fq > aln.sam

***Note:***-ax splice indicates that splicing should be taken into account, -k14 decreases the k-mer used for alignment to 14 bp which helps when aligning reads from noisy and error prone direct RNA sequencing, and -uf forces minimap2 to only attempt aligning to the forward strand since the RNA is directional. This helps increase the processing speed.

3. The outputted .sam files were converted to .bam files using samtools (http://www.htslib.org/doc/samtools-view.html)
$ samtools view -S -b

4. The resulting .bam files can be visualized in IGV.
5. TL-seq libraries were 100 bp single-end sequenced on an Illumina Hi-Seq4000.

6. Adaptors were trimmed from the sequence reads in the fastq files using cutadapt (https://cutadapt.readthedocs.io/en/stable/guide.html).
  a. Trimming the 3′ Illumina adaptor (AGATCGGAAGAGC)
    $ cutadapt -a AGATCGGAAGAGC --minimum-length=20 \
    $  -o 3prime_trimmed_file.fastq.gz \
    $  input_file.fastq.gz
  b. Using the 3′-trimmed output, the 5′ Illumina adaptor (CACTCTGAGCAATACC) was then trimmed. To select for reads that include the most 5′ end of a transcript, only carry forward reads in which the 5′ adaptor is recognized and then trimmed.
    $ cutadapt -g CACTCTGAGCAATACC --minimum-length=20 \
    $  --untrimmed-output untrimmed_output.fastq.gz \
    $  -o trimmed_output_file.fastq.gz \
    $  3prime_trimmed_file.fastq.gz

7. Reads were aligned by STAR (https://github.com/alexdobin/STAR) using indices generated from the SK1 genome assembled by combined PacBio and Illumina sequencing (https://yjx1217.github.io/Yeast_PacBio_2016/data/, Yue et al., 2017).
  $ STAR --genomeDir STAR_indices --readFilesCommand zcat \
  $  --outFileNamePrefix prefix --alignIntronMax 1 \
  $  --readFilesIn input_file.fastq.gz

8. The aligned .sam files were converted to bam files, sorted, and indexed with samtools (http://www.htslib.org/doc/samtools-view.html).
  $ samtools view -b -q 10 -o output.bam input.sam
  $ samtools sort output.bam -o output-sorted.bam
  $ samtools index output-sorted.bam

9. In R, a custom SK1 genome “SK1” was forged with BSgenome (https://bioconductor.org/packages/release/bioc/html/BSgenome.html, v1.50.0; Pagè, 2018) using the genome assembly from Yue et al. (2017) following the “How to forge a BSgenome data package” vignette.
10. TL-seq .bam files from step 8 were imported into CAGEr (http://bioconductor.org/packages/release/bioc/html/CAGEr.html, v1.24.0; Haberle et al., 2015).
  > ce <- CAGEexp(genomeName = “SK1”,
  >  inputFiles = inputFiles,
  >  inputFilesType = “bam”)

11. Reads at TSSs were counted, normalized, and initial clustering was performed.
  > getCTSS(ce, removeFirstG = FALSE)
  > normalizeTagCount(ce, method = “simpleTpm”)
  > clusterCTSS(ce, threshold = 2,
  >  thresholdIsTpm = TRUE,
  >  method = “distclu”,
  >  maxDist = 5,
  >  removeSingletons = TRUE,
  >  keepSingletonsAbove = 3)

12. The output was aggregated into larger clusters representative of all the activity expected from a single promoter.
> aggregateTagClusters(ce, tpmThreshold = 1, maxDist = 50)

13. Clustered TSSs were exported as bedGraph files for visualization in IGV.
> exportCTSStoBedGraph(ce, values = “normalized”)

14. Cluster counts were exported to DESeq2 (https://bioconductor.org/packages/release/bioc/html/DESeq2.html) by time point. Fold-changes were calculated with default settings.
  > ce$group <- factor(c(“2h”, “2h”, “2h”,
  >  “4h”, “4h”, “4h”)
  > dds <- concensusClustersDESeq2(ce, ∼group)

***Note:*** This example is for an experiment in which there were three replicates for each timepoint: 2 h in SPO (2 h) and 4 h in SPO (4 h).

15. Using the output from DESeq2, TSS clusters were filtered for coordinates in which the mean over both time points was > 2 transcripts per million and the log2 fold-change from the premeiotic stage to meiotic prophase was > 2.
16. The resulting coordinates for each meiotic prophase specific TSS were manually inputted into IGV and compared to the direct RNA sequencing reads from a sample taken during meiotic prophase.

**CRITICAL:** At each of the TSSs of interest, it was noted if at least one direct-RNA read initiated within the annotated coordinates and continued uninterrupted across the entirety of a neighboring CDS. From that subset of TSSs, potential LUTIs were confirmed if a second promoter, downstream, but on the same strand, was closer to the CDS.

17. Once LUTI candidates were defined, a secondary, more permissive clustering was performed.
  > clusterCTSS(ce, threshold = 1,
  >  thresholdIsTpm = TRUE,
  >  method = “distclu”,
  >  maxDist = 5,
  >  removeSingletons = FALSE)

***Note:*** This setting was not used initially because it resulted in an excessive number of single base pair TSSs within highly expressed genes. The TL-seq method relies on a dephosphorylation treatment prior to a decapping reaction. The decapping reaction exposes a free phosphate at the 5′-end of full-length transcripts which can then be used for the ligation of a 5′-adaptor sequence. Removing phosphates from the 5′-end of transcripts prior to decapping prevents phosphate dependent ligation of the 5′-adaptor to incomplete transcripts; however, some free 5′-phosphates will remain because the dephosphorylation reaction is not 100 % efficient. Thus, we observed low level signal across entire gene bodies at loci with high abundance transcripts. It was ideal to exclude these background signals when identifying TSS clusters. However, when quantifying abundance at the identified clusters of interest the second more permissive clustering prevented loss of signal at loci with low abundance transcripts.

18. Steps 12–14 were performed with the secondary clustering settings. The resulting values from DESeq2 were used for remaining quantification and analysis.
19. LUTI and PROX TSS were annotated to genes manually. To associate all TSS coordinates with genes, a GRanges file was created from the original SK1 genome annotation file such that there were "exon" annotations for the CDS and "promoter" annotations for the 400 bp upstream of the CDS start (taking into account the strand). This file was then used to match TSSs with genes.
  > annotateConcensusClusters(ce, GR)
  > cc <- concensusClustersGR(ce)
  > gene_df <- cc@elementMetadata
  > gene_df$coordinates <- cc@ranges@NAMES

20. A number of genes were associated with multiple TSS clusters. To determine a single most likely TSS for each gene, a custom python code was used. The jupyter notebook *single_TSS_per_gene.ipynb* can be found at https://github.com/atresen/LUTI_key_features.
21. The code to determine the single most dominant bp for each TSS (*single_bp_per_TSS.ipynb*) can be found at https://github.com/atresen/LUTI_key_features.

## Limitations

### Limitation 1

**Figure 7:**
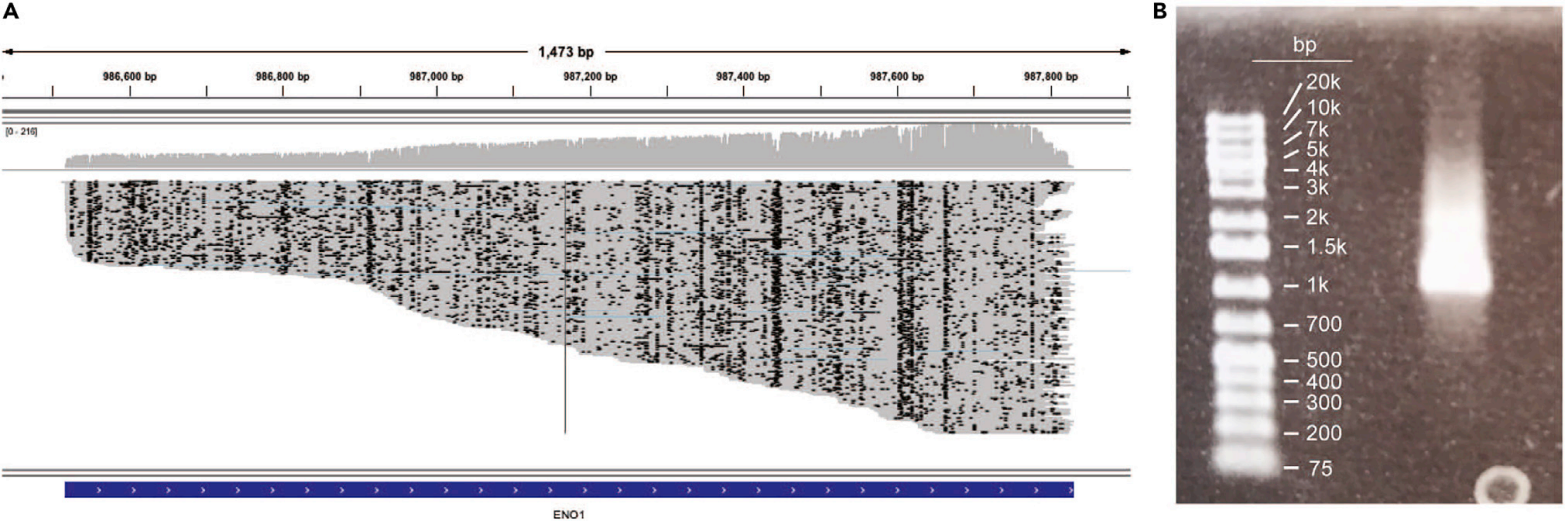
5′-truncation of control RNA in direct RNA sequencing (A) An IGV screenshot from a direct RNA sequencing run using the *ENO1* RNA control transcript (1314 base pairs) provided by ONT. Each horizontal line represents a single read. Note: Many reads that extend across the entire CDS (in blue). However, a large subset of transcripts extends over only the 3′-end of the CDS. (B) The *ENO1* control RNA on a 1 % agarose gel. Degradation of the ENO1 transcript is not apparent.

The Direct RNA Sequencing protocol produced many reads with truncated 5′-ends (Figure 7A). The truncated reads are primarily artifacts of library construction/the sequencing process (Figure 7B). As a result, at loci with LUTIs, the 5′-truncated reads cannot be defined as PROX or LUTI in origin. This limits any transcript isoform quantification using Direct RNA Sequencing.

### Limitation 2

At the *NDC80* locus, in addition to *NDC80*^*LUTI*^, evidence for a short transcript isoform initiated from the *NDC80*^*LUTI*^ promoter was previously observed (Figure 2B in Chen et al., 2017). As TL-seq only measures the abundance of 5′-transcript ends, some 5′-transcript ends mapped to distal promoters may originate from short-intergenic transcripts instead of LUTIs. Direct RNA Sequencing data can be referenced to check for the presence of short-intergenic transcripts produced by the distal promoter, but it cannot be used to estimate the proportion of LUTI:short-intergenic transcripts. This must be considered when performing quantifications by TL-seq.

### Limitation 3

The entirety of this protocol has only been tested in budding yeast of the SK1 background during meiosis. Future use of this pipeline in organisms with much larger genomes will be difficult due to the reliance on visually crosschecking TSSs from TL-seq with Direct RNA Sequencing to confirm the presence of a full-length transcript. Improvements to the computational annotation of truncated/extended transcript isoforms (i.e., isoforms that, unlike splice isoforms which harbor unique splice junctions, can only be distinguished by length) from Direct RNA Sequencing and/or another long read sequencing technique would allow this method to be transferred to a wider range of organisms.

## Troubleshooting

### Problem 1

Poor meiotic efficiency.

A meiotic efficiency rate of 90 %–95 % (determined in step 14) would be considered successful when using the copper-inducible system in this protocol. However, when starting meiosis in a new environment, it can be difficult to get all the conditions right (Figure 4).

### Potential solution

The cells are highly sensitive to water quality. We recommend washing the glassware used for growing cells without detergent. If cells are still consistently not completing meiosis at the desired efficiency, we recommend using purchased bottled water (such as Arrowhead Spring Water) to make SPO. Additionally, prototrophic strains are ideally suited for meiosis experiments whenever possible. When using an auxotrophic strain, the nutritional supplements added to SPO (adenine, uracil, histidine, leucine, and tryptophan) as described in the cell culture and media section are critical. Lastly, additional acetate (up to 2%) can enhance sporulation efficiency.

### Problem 2

Poor RNA integrity.

Partially or fully degraded RNA will reduce product yields in the final libraries. The RNA quality can be checked after total RNA has been isolated in step 23.

### Potential solution

When removing yeast cells from culture, snap freeze the pellets immediately in liquid nitrogen and store them at −80 °C. Avoid repeated freeze-thawing of extracted RNAs. Only use RNase free reagents and solutions. It is important to check the integrity of RNA inputs using an RNA Bioanalyzer or equivalent before performing this protocol. We have noticed that RNA extracted from sporulation time points tend to be lower in quality than those extracted from pre-sporulation time points. We have successfully built libraries from RNAs with RIN number > 7.

### Problem 3

Over or under-fragmentation of poly(A)+ RNAs.

The timing for fragmenting poly(A)+ RNAs to a desired size range in step 37 might vary with the sample and choice of poly(A) enrichment kit or fragmentation reagent.

### Potential solution

Test fragmentation times with a few pilot samples to determine optimal parameters for given kits. We note that fragmentation is more efficient if the input RNA had been bead or column purified.

### Problem 4

Poor enrichment of reads around TSSs.

This indicates that the dephosphorylation reaction (step 41) did not run to completion. The library is contaminated by fragments that did not originate from the capped 5′-end of transcripts.

### Potential solution

Ensure that the rSAP enzyme has been stored according to the manufacturer’s instructions. If an alternative enzyme has been used, it is critical to use the buffer, incubation time, and temperature for that enzyme.

### Problem 5

Insufficient library yields.

Poor library yields in step 62 could be due to various factors including but not limited to inefficiencies in adapter ligation, poor RNA integrity and/or decreases in enzymatic activities.

### Potential solution

Increase the amount of input material in step 24 if > 1 mg of total RNA is isolated. Increasing the number of cells collected will also increase the yield of total RNA to be used for downstream processing. Ensure the use of high quality RNA by checking the RIN of total RNA using a Bioanalyzer (step 23). Ensure that reagents, especially enzymes, are stored according to manufacturer’s instructions. The number of library PCR cycles (step 53) may need to be optimized for different library preparation kits.

### Problem 6

Too many or too few TL-seq peaks.

When reads are highly enriched around TSSs (reads observed in IGV) but output from the CAGEr program isn’t calling the expected TSS peaks in 11., 12., or is calling far more peaks than expected, the settings inputted to CAGEr should be modified.

### Potential solution

The settings presented by this protocol for clusterCTSS and aggregateTagClusters should be considered a starting point for calling peaks. The quality of 5′ transcript enrichment, the depth of sequencing, and biological factors could all alter the parameters needed to make the most ideal peak calls. Even within this protocol, two different settings were used depending on whether transcript discovery (11., 12.) or quantification (step 17) was being performed. See further discussion of this in step 17 of the quantification and statistical analysis section. When determining the settings to use for peak calling, it should be ensured that TSSs are called for a handful of transcripts that are known to be expressed at varying levels during this time. *NDC80* (LUTI and PROX), *IME2*, and *SWI4* (LUTI and PROX) are good candidates. Additionally, the utility of scanning through segments of the genome with a track of the TL-seq reads alongside a track of the called TSS peaks should not be overlooked when checking how well the peak calling algorithm predicts peaks.

## Resource availability

### Lead contact

Further information and requests for resources and reagents should be directed to and will be fulfilled by the lead contact, Elçin Ünal (elcin@berkeley.edu).

### Materials availability

The yeast strain generated for this study can be requested by contacting the lead contact.

## Data Availability

Data generated in this study are available at NCBI GEO under the accession ID GSE140177 (https://www.ncbi.nlm.nih.gov/geo/query/acc.cgi?acc=GSE140177). The custom code used for the analysis is available in the following code repository: https://github.com/atresen/LUTI_key_features.
